# Time of HIV Diagnosis and Engagement in Prenatal Care Impact Virologic Outcomes of Pregnant Women with HIV

**DOI:** 10.1371/journal.pone.0132262

**Published:** 2015-07-01

**Authors:** Florence M. Momplaisir, Kathleen A. Brady, Thomas Fekete, Dana R. Thompson, Ana Diez Roux, Baligh R. Yehia

**Affiliations:** 1 Division of Infectious Diseases and HIV Medicine, Drexel University School of Medicine, Philadelphia, Pennsylvania, United States of America; 2 AIDS Activities and Coordinating Office, Philadelphia Department of Public Health, Philadelphia, Pennsylvania, United States of America; 3 Division of Infectious Diseases, University of Pennsylvania, Philadelphia, Pennsylvania, United States of America; 4 Division of Infectious Diseases, Temple University Hospital, Philadelphia, Pennsylvania, United States of America; 5 Center for Women’s and Children’s Health Research, Christiana Care Health System, Greenville, Delaware, United States of America; 6 Drexel University School of Public Health, Philadelphia, Pennsylvania, United States of America; 7 Leonard Davis Institute of Health Economics, University of Pennsylvania, Philadelphia, Pennsylvania, United States of America; National Institute of Health, ITALY

## Abstract

**Background:**

HIV suppression at parturition is beneficial for maternal, fetal and public health. To eliminate mother-to-child transmission of HIV, an understanding of missed opportunities for antiretroviral therapy (ART) use during pregnancy and HIV suppression at delivery is required.

**Methodology:**

We performed a retrospective analysis of 836 mother-to-child pairs involving 656 HIV-infected women in Philadelphia, 2005-2013. Multivariable regression examined associations between patient (age, race/ethnicity, insurance status, drug use) and clinical factors such as adequacy of prenatal care measured by the Kessner index which classifies prenatal care as inadequate, intermediate, or adequate prenatal care; timing of HIV diagnosis; and the outcomes: receipt of ART during pregnancy and viral suppression at delivery.

**Results:**

Overall, 25% of the sample was diagnosed with HIV during pregnancy; 39%, 38%, and 23% were adequately, intermediately, and inadequately engaged in prenatal care. Eight-five percent of mother-to-child pairs received ART during pregnancy but only 52% achieved suppression at delivery. Adjusting for patient factors, pairs diagnosed with HIV during pregnancy were less likely to receive ART (AOR 0.39, 95% CI 0.25-0.61) and achieve viral suppression (AOR 0.70, 95% CI 0.49-1.00) than those diagnosed before pregnancy. Similarly, women with inadequate prenatal care were less likely to receive ART (AOR 0.06, 95% CI 0.03-0.11) and achieve viral suppression (AOR 0.31, 95% CI 0.20-0.47) than those with adequate prenatal care.

**Conclusions:**

Targeted interventions to diagnose HIV prior to pregnancy and engage HIV-infected women in prenatal care have the potential to improve HIV related outcomes in the perinatal period.

## Introduction

HIV viral suppression during pregnancy and at the time of delivery is crucial to preventing mother to child transmission (MTCT) of HIV.[1–4] Among untreated mothers, 70% of HIV transmissions occur during labor and delivery, [5, 6] which represent a critical window period where interventions to optimize viral suppression are essential. In the U.S., public health recommendations to prevent MTCT of HIV include: (1) universal HIV screening during the first trimester, (2) use of combination antiretroviral therapy (ART) among all HIV-infected pregnant women regardless of viral load (VL) or CD4 count, (3) delivery via scheduled Caesarean section when maternal VL is >1,000 copies/ml, and (4) post-exposure prophylaxis for all HIV-exposed infants.[7, 8] Implementation of these recommendations have successfully reduced the incidence of HIV vertical transmission in the U.S. from 22% in the pre-ART era[4] to 2% in the modern ART era[9]. Despite this success, MTCT persists and is particularly elevated among ethnic minorities.[10] There are approximately 278,000 women age 13 or older living with HIV in the US and the number of women with HIV giving birth has increased 30% more from 6,000 in 2000 to 8,700 in 2006.[10] These findings suggest that additional efforts are needed to optimize maternal HIV care in order to prevent vertical transmission of HIV and reach the goal of <1% MTCT rate established by the Centers for Disease Control (CDC)[11].

The benefits of viral suppression during pregnancy go beyond the reduction of MTCT; the impact of uncontrolled HIV disease has direct implications on women and fetal health and increases the economic burden on communities. Uncontrolled viral replication and ART non-adherence lead to viral resistance, AIDS, and is associated with a higher risk of HIV transmission. Vis-à-vis the infant, fetal health is directly linked to maternal health. Studies show that uninfected but HIV-exposed infants born to HIV-infected mothers have higher rates of mortality than do infants born to uninfected mothers, and infant mortality is associated with advanced maternal HIV disease.[12–14] Data from 7,638 HIV exposed but uninfected infants in France show a 60% increase risk of serious bacterial infection when maternal CD4 is <350 cells/mm^3^.[15] Quantitative and qualitative immunological studies have demonstrated that impaired maternal humoral immunity, which is passively transferred through the placenta, result in reduced infant CD4/CD8,[16] naïve or memory T cells,[16, 17] cytokine production and increased T cell apoptosis.[16, 18]

Use of ART and HIV viral suppression during pregnancy are achievable goals since many pregnant women are motivated to take ART to minimize the risk of MTCT.[19, 20] As such, early HIV diagnosis and engagement in prenatal care are modifiable factors that have the potential of improving viral suppression at delivery. To ensure that HIV-infected pregnant women receive all available HIV prevention services, public health authorities need to understand factors associated with missed opportunities of ART receipt during pregnancy and viral suppression at delivery. Our aim was to evaluate how timing of HIV diagnosis and engagement in prenatal care are associated with receipt of ART during pregnancy and viral suppression at delivery. We hypothesized that women diagnosed with HIV during pregnancy and those with inadequate engagement in prenatal care would be less likely to receive ART during pregnancy and less likely to be suppressed at delivery.

## Materials and Methods

### Study Population

The Enhanced Perinatal Surveillance (EPS) project is a population-based surveillance system of HIV-infected pregnant women in 15 high incidence areas, including 9 U.S. states, 5 U.S. cities, and Puerto Rico. The overarching goals of EPS are to assist public health officials in timely evaluation of perinatal HIV prevention efforts and assess the use of ART among HIV-infected pregnant women. Mother and infant pairs are identified through comprehensive epidemiologic surveillance methods described elsewhere.[21] In brief, mother and infant pairs are identified through the pediatric HIV surveillance system, laboratory reporting, reports of HIV-infected pregnant women, hospital discharge summaries, and matches of HIV-infected women with vital statistics birth registry data. Health departments in designated areas collect demographic and clinical information on HIV-infected mothers including engagement in prenatal care, HIV testing history, and use of ART mainly by means of chart abstraction.

We performed a retrospective analysis using the Philadelphia EPS, which includes all HIV-infected women who delivered a live infant in Philadelphia and the surrounding counties between January 2005 and May 2013. EPS was merged with the Enhanced HIV/AIDS Reporting System (eHARS). eHARS is a surveillance system of all reported HIV/AIDS cases in Pennsylvania that is conducted by the Pennsylvania Department of Public Health in cooperation with the Philadelphia Department of Public Health. eHARS contains demographic and clinical information of HIV-infected individuals including all HIV VL.

### Outcome Variables

Our outcomes of interest were receipt of ART during pregnancy and viral suppression at delivery. Receipt of ART was defined as having at least three antiretrovirals prescribed at any point during the pregnancy. HIV viral suppression was defined as having an HIV VL ≤ 400 copies/ml at the time closest to the delivery date up to 30 days postpartum. The cut-off value of 400 copies/ml was used in the early years of our study period and applied to the entire cohort for consistency. When missing, the VLs were supplemented with eHARS VLs.

### Independent Variables

Demographic variables included age, race/ethnicity, and health insurance status. Age was classified as 16–24, 25–34, and ≥35 years; race/ethnicity was grouped as white non-Hispanic, black non-Hispanic, Hispanic/Latino, and other. Health insurance status was classified as public, private, or uninsured. Public insurance included patients on Medicaid, Medicare, and other state funding. Use of illicit drugs during pregnancy was determined through medical and social work chart review and toxicology results. Engagement in prenatal care was assessed using a widely accepted, validated index of adequacy of prenatal care—the Kessner Institute of Medicine Index. This measure takes into account timing of entry in prenatal care, number of prenatal visits, and gestational age at delivery. [22] The timing of HIV diagnosis was classified as before or during pregnancy. Year of infant delivery was grouped in three evenly split time periods: 2005–2007, 2008–2010 and 2011–2013. In each time period, major revisions in ART treatment took place: in 2007, guidelines revisions called for initiation of ART when the CD4 was < 350 cell/μl; in 2009, the CD4 count treatment cut off level moved to 500 and in 2012, ART was to be initiated among all HIV-infected individuals, regardless of CD4 count.

### Statistical Analysis

The analysis included each delivery and was therefore performed at the mother-to-child pair level. We first evaluated the progression of all demographic, clinical and outcome variables over the previously described time periods: 2005–2007, 2008–2010 and 2011–2013. We then compared sociodemographic and clinical differences in the proportion of mother-to-child pairs on ART (versus not) during pregnancy and virally suppressed (versus not) at delivery using Pearson χ^2^ tests. Multivariable logistic regression was used to estimate the association between sociodemographic (age, race, insurance status, drug use) and clinical (adequacy of prenatal care and timing of HIV diagnosis) factors and the two outcomes, receipt of ART during pregnancy and viral suppression at delivery. STATA 12 (StataCorp, College Station, TX) was used for data analysis.

The data generated for this study were subjected to the same security and confidentiality requirements as the national EPS. This includes adherence to CDC guidelines for the security and confidentiality of HIV/AIDS surveillance data. IRB exemption was granted by the Philadelphia Department of Public Health. Written consent was not obtained for this study. Patient records were anonymized and de-identified prior to data analysis.

## Results

Our cohort included 836 mother-to-child pairs involving 656 HIV-infected women between 2005 and 2013. There were 12% missing VLs. Missing VLs were grouped with the unsuppressed (HIV VL > 400 copies/ml) since a sensitivity analysis after exclusion of the missing VLs did not substantially alter findings of our data analysis. The majority (82%) of VLs were obtained during the second or third trimesters with a mean of 8 weeks (SD 9.2) before delivery (the 50^th^ percentile was -5 weeks).

Of all the mother-to-child pairs, 51% were between 25 and 34 years old, 79% were black non-Hispanic, 78% were publically insured, and 23% used illicit drugs during pregnancy (Table 1). In total, 39%, 38%, and 23% of mother-to-child pairs were adequately, intermediately, and inadequately engaged in prenatal care, respectively. Three quarters were diagnosed with HIV before pregnancy and a quarter were diagnosed during pregnancy. Most mother-to-child pairs (85%) received ART during pregnancy and only 52% achieved viral suppression at delivery (Table 1). Among the 836 mother-to-child pairs, 18 infants contracted HIV resulting in a MTCT rate of 2.1%.

**Table 1:** Demographic, Behavioral and Quality of Care Indicators among Pregnant Women Infected with HIV, Enhanced Perinatal Surveillance Project, Philadelphia, 2005–2013.

	Total, n (%) n = 836	2005–2007	2008–2010	2011–2013	p- value
**Demographic/Behavioral**					
**Age (years)**					0.29
16–24	214 (25.6)	97 (28.0)	71 (23.1)	46 (25.3)	
25–34	431 (51.6)	180 (52.0)	164 (53.2)	87 (47.8)	
≥35	191 (22.8)	69 (20.0)	73 (23.7)	49 (26.9)	
**Race/Ethnicity**					0.36
White, non-Hispanic	72 (8.6)	36 (10.4)	25(8.1)	11 (6.0)	
Black, non-Hispanic	661 (79.1)	271 (78.3)	244 (79.2)	146 (80.2)	
Hispanic or Latino	68 (8.1)	24 (6.9)	24 (7.8)	20 (11.0)	
Other	35 (4.2)	15 (4.3)	15 (4.9)	5 (2.8)	
**Insurance**					<0.001
Public	650 (77.7)	290 (83.8)	232 (75.3)	128 (70.3)	
Private	97 (11.6)	30 (8.7)	46 (14.9)	21 (11.5)	
Uninsured	89 (10.6)	26 (7.5)	30 (9.7)	33 (18.1)	
**Drug Use During Pregnancy**					0.67
Yes	191 (22.8)	76 (22.0)	69 (22.4)	46 (25.3)	
No	645 (77.2)	270 (78.0)	239 (77.6)	136 (74.7)	
**Quality of Care Indicators**					
**Adequacy of Prenatal Care** †					0.006
Adequate	325 (38.9)	153 (44.2)	108 (35.1)	64 (35.2)	
Intermediate	319 (38.2)	113 (32.7)	120 (39.0)	86 (47.2)	
Inadequate	192 (23.0)	80 (23.1)	80 (26.0)	32 (17.6)	
**Timing of HIV Diagnosis**					0.040
Before Pregnancy	625 (74.8)	243 (70.2)	241 (78.3)	141 (77.5)	
During Pregnancy	211 (25.3)	103 (29.8)	67 (21.7)	41 (22.5)	
**Receipt of ART** *					0.001
Yes	708 (84.7)	276 (79.8)	264 (85.7)	168 (92.3)	
No	128 (15.3)	70 (20.2)	44 (14.3)	14 (7.7)	
**Viral Suppression at Delivery** **					<0.001
Yes	437 (52.3)	123 (35.6)	189 (61.4)	125 (68.7)	
No	399 (47.7)	223 (64.4)	119 (38.6)	57 (31.3)	

*ART = Antiretroviral Therapy. 708 (84.69%) mother-to-child pairs received ART during pregnancy.

**Viral suppression is defined as having a VL(viral load) ≤400 copies/ml. 437 (52.27%) mother-to-child pairs achieved viral suppression at delivery. The HIV VLs > 400 copies/ml include 12.44% missing HIV VL. The VLs were measured during pregnancy and up to 30 days postpartum.

^†^ Adequacy of prenatal care was measured using the Kessner Index which takes in account timing of entry in prenatal care, the number of prenatal visits and the gestational age at delivery. It is a validated measure of quality of prenatal care.

The age, race/ethnicity and drug use among mother-to-child pairs did not vary over time. The uninsured increased as the number of mother-to-child pairs publically insured decreased. Adequacy of prenatal care and the timing of HIV diagnosis improved as more mother-to-child pairs received less inadequate care and more intermediate care and more HIV diagnoses were established before as opposed to during pregnancy (Table 1). Receipt of ART and viral suppression significantly improved as well: 80% of mother-to-child pairs received ART in 2005–2007, that proportion increased to 92% in 2011–2013; 36% of mother-to-child pairs were suppressed at delivery in 2005–2007 as opposed to 69% in 2011–2013. In our multivariate analysis, pairs with an infant delivery in 2011–2013 were 3 times more likely to receive ART and 4 times more likely to achieve suppression compared to pairs with an infant delivery in 2005–2007.

Mother-to-child pairs receiving and not receiving ART during pregnancy were similar in age, race/ethnicity, and health insurance status (S1 Table). However, pairs on ART were less likely to use illicit drugs during pregnancy (20% vs. 40%), more likely to engage in adequate prenatal care (44% vs. 12%), and more likely to be diagnosed with HIV before pregnancy (79% vs. 53%) than the pairs not on ART. Other than drug use, demographic factors were similar among those who achieved suppression at delivery versus those who did. Mother-to-child pairs with a suppressed VL at delivery were more likely to have received adequate prenatal care (45% vs. 32%) and have an HIV diagnosis before pregnancy (80% vs. 69%) compared to pairs with unsuppressed VL at delivery.

**Fig 1:**
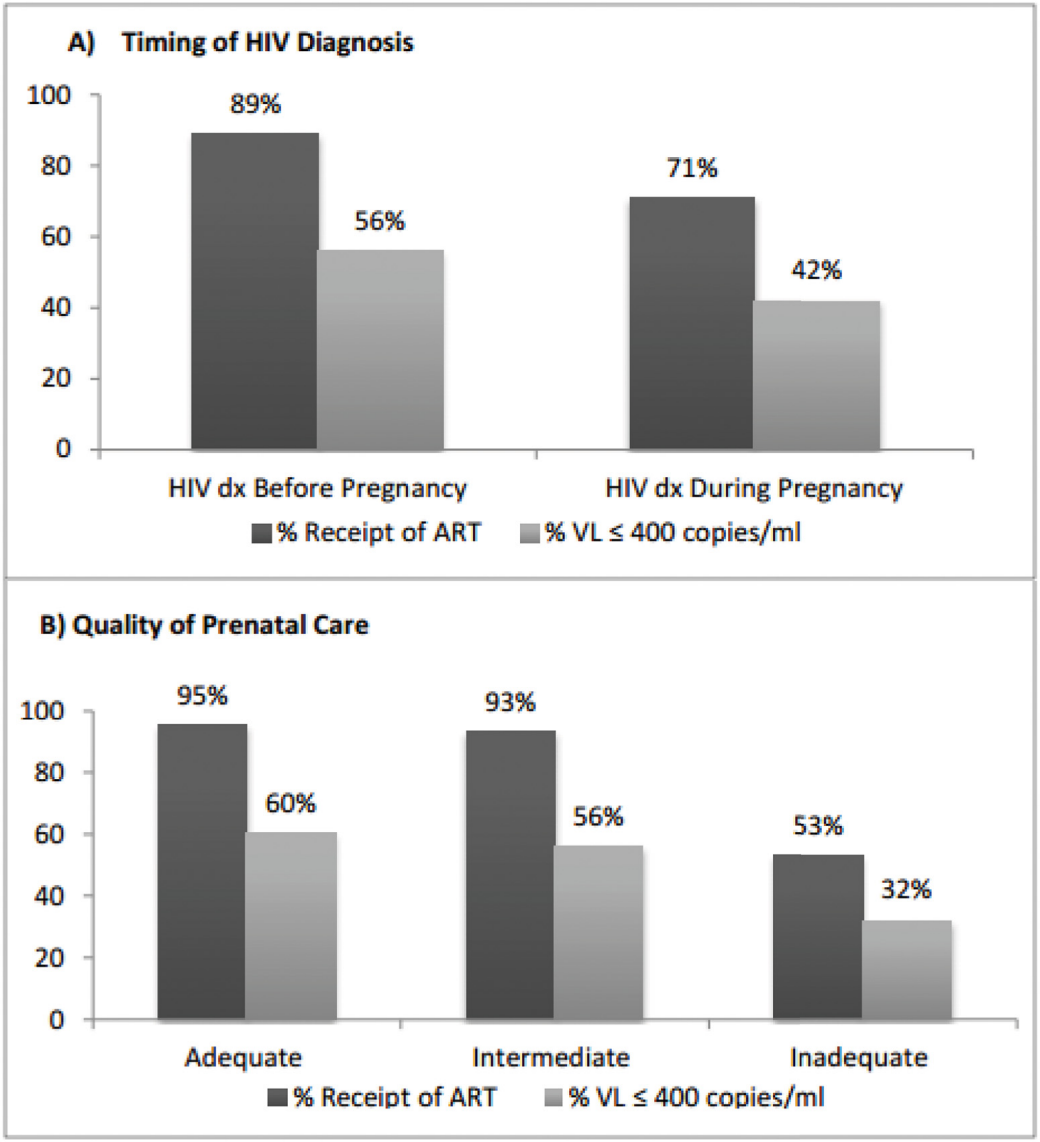
Receipt of ART and Viral Suppression by A) Timing of HIV Diagnosis and B) Quality of Prenatal Care. Adequacy of prenatal care was measured using the Kessner Index, a validated index of quality of prenatal care which takes into account timing of entry in prenatal care, the number of prenatal visits and gestational age at infant delivery; ART: antiretroviral; VL: viral load; dx: diagnosis.

Outcomes differed by timing of HIV diagnosis and engagement in prenatal care. Overall, 89% of mother-to-child pairs diagnosed with HIV before pregnancy received ART and 56% achieved viral suppression at delivery. In contrast, 71% of pairs diagnosed with HIV during pregnancy received ART and only 42% achieved viral suppression at delivery (Fig 1). When considering engagement in prenatal care, 95% of mother-to-child pairs with adequate care engagement received ART and 60% achieved suppression. Corresponding proportions for those intermediately and inadequately engaged in care were 93% and 56% and 53% and 32%, respectively. In multivariate logistic regression models, engagement in prenatal care, timing of HIV diagnosis, and infant birth year were significantly associated with both ART receipt and viral suppression (Table 2). Mother-to-child pairs with inadequate prenatal care were less likely to receive ART (AOR 0.06, 95% CI 0.03–0.11) and achieve viral suppression (AOR 0.31, 95% CI 0.20–0.47) compared to those with adequate prenatal care. The likelihood of ART receipt (AOR 0.39, 95% CI 0.25–0.61) and viral suppression (AOR 0.70, 95% CI 0.49–1.00) were lower among mother-to-child pairs diagnosed with HIV during compared to before pregnancy.

**Table 2:** Demographic, Behavioral and Clinical Factors associated with 1) ART Prescription and 2) HIV Viral Suppression using Multivariate Logistic Regression.

	Receipt of ART	Viral Suppression
	AOR (95% CI)	AOR (95% CI)
16–24 (years)	-	-
25–34	**0.48 (0.26–0.88)**	0.98 (0.68–1.44)
≥ 35	**0.42 (0.20–0.87)**	0.88 (0.56–1.40)
White, non-Hispanic	-	-
Black, non-Hispanic	0.48 (0.19–1.20)	0.74 (0.44–1.25)
Hispanic or Latino	0.33 (0.10–1.10)	0.72 (0.35–1.51)
Other	0.91 (0.22–3.75)	0.62 (0.23–1.71)
Public Insurance	-	-
Private Insurance	0.82 (0.37–1.80)	1.18 (0.71–1.96)
Uninsured	1.19 (0.54–2.60)	1.34 (0.78–2.31)
Illicit Drug Use, No	-	-
Illicit Drug Use, Yes	0.75 (0.45–1.28)	0.77 (0.53–1.13)
Adequate Prenatal Care	-	-
Intermediate Prenatal Care	0.62 (0.31–1.24)	0.74 (0.53–1.04)
Inadequate Prenatal Care	**0.06 (0.03–0.11)**	**0.31 (0.20–0.47)**
HIV Dx Before Pregnancy	-	-
HIV Dx During Pregnancy	**0.39 (0.25–0.61)**	**0.70 (0.49–1.00)**
Year of Delivery 2005–2007	-	-
Year of Delivery 2008–2010	**2.07 (1.23–3.48)**	**3.19 (2.27–4.49)**
Year of Delivery 2011–2013	**3.60 (1.82–7.14)**	**4.13 (2.75–6.20)**

ART—antiretroviral; Dx—diagnosis; CI—confidence interval; bold face font, statistically significant p < 0.001.

## Discussion

Our study is the first to use population based data to evaluate viral suppression rates of HIV-infected pregnant women at delivery. Our findings have significant public health implications for HIV, maternal and fetal health and thus provide a platform for future public health interventions. We found that 85% of mother-to-child pairs received ART during pregnancy but only half achieved viral suppression at delivery. These viral suppression rates, although lower than those reported elsewhere, [23–25] were collected in undisturbed, real life settings, and directly reflect patterns of HIV suppression at delivery from a cohort representative of HIV-infected pregnant women in a major U.S city. Factors associated with both receipt of ART and viral suppression included timing of HIV diagnosis, quality of prenatal care and birth year.

In the Women and Infants Transmission Study (WITS), women had a viral suppression rate of 68% at delivery; [23, 26] however, WITS is by design a research cohort where women are actively enrolled and followed up upon with use of detailed medical and behavioral questionnaires. In addition, the study was conducted a decade ago (between 1998 and 2005); the choice and availability of ART in the general population and among pregnant women, as well as policies relating to HIV care, have evolved since then. Data from a recent analysis of the International Maternal, Pediatric, Adolescent AIDS Clinical Trials (IMPAACT) Group, 2002–2011, reported that 13% of women who newly initiated ART during pregnancy had a detectable VL at delivery. Women with no reported use of ART during pregnancy and those with ART use prior to conception were excluded from the study[25]. Our analysis of population based data allowed the inclusion of women in various clinical settings (university associated clinics, private practice or community health centers) and showed that the rate of viral suppression among HIV-infected pregnant is much lower than anticipated. This finding has direct implications on maternal and fetal health since maternal immunosuppression is closely linked to maternal and fetal morbidity. Beyond the goal of reducing mother-to-child transmission, our findings call for additional public health interventions to improve the quality of HIV care in the perinatal period.

Our study adds to the existing literature by identifying women at high risk of virological failure at delivery. In our univariate analysis, women with active drug use, with inadequate prenatal care, with a late HIV diagnosis (i.e. HIV diagnosis made during instead of before pregnancy) were less likely to receive ART and be suppressed at delivery. Our findings are in accordance with prior data showing that late initiation of ART and delayed entry into prenatal care were significantly associated with a detectable viral load at delivery[25]. Women with poor engagement in prenatal care are at high-risk of clinical failure during the perinatal periods and should be identified as such during routine care. In addition, measures to optimize viral suppression at delivery should be applied. The role of directly observed therapy and raltegravir intensification has been shown to be beneficial in other populations [27, 28] and should be evaluated among HIV-infected women with poor engagement in prenatal care and poor adherence to ART.

In our multivariate analysis, an HIV diagnosis made during pregnancy was strongly associated with poor receipt of ART and viral suppression. This is in contrast with results from the IMPAACT Group showing that a slightly larger proportion of women diagnosed with HIV before pregnancy had detectable VL at delivery compared to women diagnosed with HIV during pregnancy (16% versus 11%, p = 0.05). Timing of HIV diagnosis was not included in their multivariate logistic regression model, it’s unclear if this relationship would remain after adjustment of confounders[25]. An HIV diagnosis made before pregnancy allows time for patients and providers to work through treatment decisions and life adjustments. This process is even more important for women with repeated pregnancies as data show that multiparous women are less likely to adhere to ART because, among other things, the increased child care burden.[25, 29] The implementation of opt-out HIV testing in all healthcare settings as advocated by the CDC and the US Preventive Services Task Force is likely to result in early diagnosis of HIV among women of child bearing age. HIV testing needs to also be performed outside of traditional healthcare settings to reach women who are out of care and who are at risk of unplanned pregnancies, particularly those who exchange money for drugs or sex. Women diagnosed with HIV during pregnancy should receive supportive services tailored to their emotional needs (particularly with HIV acceptance and disclosure) and clinical needs in order to improve their health outcomes.

Women who received inadequate prenatal care were less likely to adhere to therapy and achieve viral suppression at delivery. Surely, adherence to prenatal visits is a surrogate marker of women’s motivation to achieve good health outcomes for themselves and their infants and facilitates repeated assessments of HIV disease. According to the American Congress of Obstetricians and Gynecologists prenatal care guidelines,[30] HIV infected women should engage in care during their first trimester, have an average of 11 prenatal visits during pregnancy and have frequent viral load monitoring if adherence to ART is a concern. Studies have shown that poor engagement in prenatal care predict maternal loss to follow-up post-partum and negatively affect MTCT rates.[31, 32] Possible solutions to improve the quality of prenatal care include identifying women with repeated missed visits and use of interventions which have been proven to work in other settings such as the peer-coaching,[33–35] financial incentives,[36, 37] and the involvement of case management,[38, 39] particularly for women who are homeless or suffer from mental health and substance abuse.

Other documented barriers to ART adherence and prenatal care not measured in this study include stigma,[19, 40, 41] socioeconomic stressors, [19, 41–43] lack of social support,[20, 41, 42] and mental health.[20, 44] Qualitative studies are needed to understand how socio-cognitive and socio-contextual factors influence women’s decision making vis-à-vis HIV care engagement during pregnancy. Health system factors, such as models of HIV care delivery where HIV and obstetrical care and offered at the same site versus separate sites, and their association with virological failure at delivery need to be evaluated. For optimal maternal and fetal health outcomes, HIV, obstetrical and pediatric care need to be closely coordinated.

Despite overall low viral suppression rates, we found that ART use and viral suppression significantly improved over time. During our study period, the Department of Health and Human Services guidelines for initiation of ART in the general population changed multiple times, and in each instance the initiation of ART was recommended at higher CD4 counts.[45] The improved ART receipt and viral suppression rates observed during our study period could be related to these guideline revisions as well as other unmeasured factors.

The strengths of our study include its sample size. We captured 836 deliveries over a 9 year period. Because of mandatory reporting of HIV infected mothers and their perinatally exposed infants, we were able to capture the vast majority of women with HIV who had a delivery in Philadelphia and its surrounding counties. The Philadelphia Department of Public Health case ascertainment is estimated to be 98.5% using CDC supplied programs. Our analysis was also done using real life data in undisturbed clinical settings and is representative of the population studied. The limitations include shortcomings associated with secondary data analysis. Since the majority of variables other than laboratory values were obtained from chart abstraction, we depend on the quality of documentation from the providers during the prenatal visits and hospitalizations. This is particularly relevant to the ART receipt variable since we had no complementary pharmacy data available to verify receipt of ART. Important variables, such as a diagnosis of mental health, intimate partner violence and planned versus unplanned pregnancy were not captured in our database. We would caution against extrapolations of our findings to populations in cities with differences in local resources, demography and behavioral trends during pregnancy.

In conclusion, we found that a large proportion of pregnant women infected with HIV failed to achieve viral suppression at delivery. Even though viral suppression improved over the time period of the study, women diagnosed with HIV during pregnancy and who received inadequate prenatal care were significantly less likely to be suppressed at delivery. These women, because of their risk for poor HIV outcomes, should be seen as a vulnerable group in which women-centered, culturally sensitive outreach measures should be implemented as early as possible during pregnancy in order to avoid complications related to uncontrolled HIV disease for the mother and the infant.

## Supporting Information

S1 Table *ART = Antiretroviral Therapy. 708 (84.69%) mother-to-child pairs received ART during pregnancy. **VL = viral load. 437 (52.27%) mother-to-child pairs achieved viral suppression at delivery. The HIV VL > 400 copies/ml includes 12.44% missing HIV VL. The VL were measured during pregnancy and up to 30 days postpartum. † Adequacy of prenatal care was measured using the Kessner Index which takes in account timing of entry in prenatal care, the number of prenatal visits and the gestational age at delivery. It is a validated measure of quality of prenatal care.
(DOCX)

## References

[1] Warszawski J, Tubiana R, Le Chenadec J, Blanche S, Teglas J-P, Dollfus C, et al Mother-to-child HIV transmission despite antiretroviral therapy in the ANRS French Perinatal Cohort. Aids. 2008;22(2):289–99. 18097232 10.1097/QAD.0b013e3282f3d63c

[2] Chasela CS, Hudgens MG, Jamieson DJ, Kayira D, Hosseinipour MC, Kourtis AP, et al Maternal or infant antiretroviral drugs to reduce HIV-1 transmission. New England Journal of Medicine. 2010;362(24):2271–81. 10.1056/NEJMoa0911486 20554982 PMC3440865

[3] Dickover RE, Garratty EM, Herman SA, Sim M-S, Plaeger S, Boyer PJ, et al Identification of levels of maternal HIV-1 RNA associated with risk of perinatal transmission: effect of maternal zidovudine treatment on viral load. Jama. 1996;275(8):599–605. 8594240

[4] Sperling RS, Shapiro DE, Coombs RW, Todd JA, Herman SA, McSherry GD, et al Maternal viral load, zidovudine treatment, and the risk of transmission of human immunodeficiency virus type 1 from mother to infant. New England Journal of Medicine. 1996;335(22):1621–9. 8965861 10.1056/NEJM199611283352201

[5] De Cock KM, Fowler MG, Mercier E, de Vincenzi I, Saba J, Hoff E, et al Prevention of mother-to-child HIV transmission in resource-poor countries: translating research into policy and practice. Jama. 2000;283(9):1175–82. 10703780 10.1001/jama.283.9.1175

[6] Simonon A, Lepage P, Karita E, Hitimana D-G, Dabis F, Msellati P, et al An assessment of the timing of mother-to-child transmission of human immunodeficiency virus type 1 by means of polymerase chain reaction. JAIDS Journal of Acquired Immune Deficiency Syndromes. 1994;7(9):952–7. 8051621

[7] Panel on Treatment of HIV-Infected Pregnant Women and Prevention of Perinatal Transmission. Recommendations for Use of Antiretroviral Drugs in Pregnant HIV-1-Infected Women for Maternal Health and Interventions to Reduce Perinatal HIV Transmission in the United States. Available at http://aidsinfo.nih.gov/contentfiles/lvguidelines/PerinatalGL.pdf. Accessed July 9, 2014.

[8] Branson BM, Handsfield HH, Lampe MA, Janssen RS, Taylor AW, Lyss SB, et al Revised recommendations for HIV testing of adults, adolescents, and pregnant women in health-care settings: US Department of Health and Human Services, Centers for Disease Control and Prevention; 2006. 16988643

[9] Whitmore SK, Taylor AW, Espinoza L, Shouse RL, Lampe MA, Nesheim S. Correlates of mother-to-child transmission of HIV in the United States and Puerto Rico. Pediatrics. 2012;129(1):e74–e81. 10.1542/peds.2010-3691 22144694

[10] Centers for Disease Control and Prevention. http://www.cdc.gov/hiv/risk/gender/pregnantwomen/. Last accessed June 1, 2014.

[11] Nesheim S, Taylor A, Lampe MA, Kilmarx PH, Harris LF, Whitmore S, et al A framework for elimination of perinatal transmission of HIV in the United States. Pediatrics. 2012;130(4):738–44. 10.1542/peds.2012-0194 22945404

[12] Chilongozi D, Wang L, Brown L, Taha T, Valentine M, Emel L, et al Morbidity and mortality among a cohort of human immunodeficiency virus type 1-infected and uninfected pregnant women and their infants from Malawi, Zambia, and Tanzania. The Pediatric infectious disease journal. 2008;27(9):808 10.1097/INF.0b013e31817109a4 18679152 PMC2739309

[13] Kuhn L, Kasonde P, Sinkala M, Kankasa C, Semrau K, Scott N, et al Does severity of HIV disease in HIV-infected mothers affect mortality and morbidity among their uninfected infants? Clinical Infectious Diseases. 2005;41(11):1654–61. 16267740 10.1086/498029 PMC1351118

[14] Zaba B, Whitworth J, Marston M, Nakiyingi J, Ruberantwari A, Urassa M, et al HIV and mortality of mothers and children: evidence from cohort studies in Uganda, Tanzania, and Malawi. Epidemiology. 2005;16(3):275–80. 15824540 10.1097/01.ede.0000155507.47884.43

[15] Taron-Brocard C, Le Chenadec J, Faye A, Dollfus C, Goetghebuer T, Gajdos V, et al Increased risk of serious bacterial infections due to maternal immunosuppression in HIV-exposed uninfected infants in a European country. Clinical Infectious Diseases. 2014;59(9):1332–45. 10.1093/cid/ciu586 25053719

[16] Clerici M, Saresella M, Colombo F, Fossati S, Sala N, Bricalli D, et al T-lymphocyte maturation abnormalities in uninfected newborns and children with vertical exposure to HIV. Blood. 2000;96(12):3866–71. 11090071

[17] Romano MF, Buffolano W, Bisogni R, Russo R, Liuzzi R, Bunders M, et al Increased CD154 expression in uninfected infants born to HIV-positive mothers exposed to antiretroviral prophylaxis. Viral immunology. 2006;19(3):363–72. 16987056 10.1089/vim.2006.19.363

[18] Economides A, Schmid I, Anisman-Posner DJ, Plaeger S, Bryson YJ, Uittenbogaart CH. Apoptosis in cord blood T lymphocytes from infants of human immunodeficiency virus-infected mothers. Clinical and diagnostic laboratory immunology. 1998;5(2):230–4. 9521148 10.1128/cdli.5.2.230-234.1998 PMC121363

[19] Ngarina M, Popenoe R, Kilewo C, Biberfeld G, Ekstrom AM. Reasons for poor adherence to antiretroviral therapy postnatally in HIV-1 infected women treated for their own health: experiences from the Mitra Plus study in Tanzania. BMC public health. 2013;13(1):450. 23647555 10.1186/1471-2458-13-450 PMC3651864

[20] Nachega JB, Uthman OA, Anderson J, Peltzer K, Wampold S, Cotton MF, et al Adherence to antiretroviral therapy during and after pregnancy in low-income, middle-income, and high-income countries: a systematic review and meta-analysis. Aids. 2012;26(16):2039–52. 10.1097/QAD.0b013e328359590f 22951634 PMC5061936

[21] Centers_for_Disease_Control_and_Prevention. Enhanced perinatal surveillance—15 areas, 2005–2008. HIV Surveillance Supplemental Report 2011 http://www.cdc.gov/hiv/topics/surveillance/resources/reports/ [cited 2011 April 2014].

[22] Kessner DM, Singer J, Kalk CE, Schlesinger ER. Infant Death: An Analysis by Maternal Risk and Health Care Contrasts in Health Status. Vol. I Washington, DC Institute of Medicine. National Academy of Sciences 1973.

[23] Cooper ER, Charurat M, Mofenson L, Hanson IC, Pitt J, Diaz C, et al Combination antiretroviral strategies for the treatment of pregnant HIV-1-infected women and prevention of perinatal HIV-1 transmission. Journal of acquired immune deficiency syndromes (1999). 2002;29(5):484–94. 11981365 10.1097/00126334-200204150-00009

[24] Yehia BR, Schranz AJ, Momplaisir F, Keller SC, Gross R, Frank I, et al Outcomes of HIV-Infected Patients Receiving Care at Multiple Clinics. AIDS and behavior. 2013:1–12. 10.1007/s10461-012-0321-z 24077931 PMC3969411

[25] Katz IT, Leister E, Kacanek D, Hughes MD, Bardeguez A, Livingston E, et al Factors Associated With Lack of Viral Suppression at Delivery Among Highly Active Antiretroviral Therapy–Naive Women With HIV: A Cohort Study. Annals of internal medicine. 2015;162(2):90–9. 10.7326/M13-2005 25599347 PMC4299931

[26] Katz IT, Shapiro R, Li D, Govindarajulu U, Thompson B, Watts DH, et al Risk factors for detectable HIV-1 RNA at delivery among women receiving highly active antiretroviral therapy in the women and infants transmission study. Journal of acquired immune deficiency syndromes (1999). 2010;54(1):27. 20065861 10.1097/QAI.0b013e3181caea89 PMC2860013

[27] Hatano H, Hayes TL, Dahl V, Sinclair E, Lee T-H, Hoh R, et al A randomized, controlled trial of raltegravir intensification in antiretroviral-treated, HIV-infected patients with a suboptimal CD4+ T cell response. Journal of Infectious Diseases. 2011;203(7):960–8. 10.1093/infdis/jiq138 21402547 PMC3068029

[28] Binford MC, Kahana SY, Altice FL. A systematic review of antiretroviral adherence interventions for HIV-infected people who use drugs. Current HIV/AIDS Reports. 2012;9(4):287–312. 10.1007/s11904-012-0134-8 22936463 PMC3495269

[29] Merenstein D, Schneider MF, Cox C, Schwartz R, Weber K, Robison E, et al Association of child care burden and household composition with adherence to highly active antiretroviral therapy in the Women's Interagency HIV Study. AIDS patient care and STDs. 2009;23(4):289–96. 10.1089/apc.2008.0161 19243274 PMC2674283

[30] American College of Obstetrics and Gynecology http://www.acog.org/Last accessed May, 2015.

[31] Townsend CL, Byrne L, Cortina-Borja M, Thorne C, de Ruiter A, Lyall H, et al Earlier initiation of ART and further decline in mother-to-child HIV transmission rates, 2000–2011. AIDS. 2014;28(7):1049–57. 10.1097/QAD.0000000000000212 24566097

[32] Siddiqui R, Bell T, Sangi-Haghpeykar H, Minard C, Levison J. Predictive Factors for Loss to Postpartum Follow-Up Among Low Income HIV-Infected Women in Texas. AIDS patient care and STDs. 2014;28(5):248–53. 10.1089/apc.2013.0321 24720630

[33] Long JA, Jahnle EC, Richardson DM, Loewenstein G, Volpp KG. Peer Mentoring and Financial Incentives to Improve Glucose Control in African American VeteransA Randomized Trial. Annals of internal medicine. 2012;156(6):416–24. 10.7326/0003-4819-156-6-201203200-00004 22431674 PMC3475415

[34] Purcell DW, Latka MH, Metsch LR, Latkin CA, Gómez CA, Mizuno Y, et al Results from a randomized controlled trial of a peer-mentoring intervention to reduce HIV transmission and increase access to care and adherence to HIV medications among HIV-seropositive injection drug users. JAIDS Journal of Acquired Immune Deficiency Syndromes. 2007;46:S35–S47. 18089983 10.1097/QAI.0b013e31815767c4

[35] Naar-King S, Outlaw A, Green-Jones M, Wright K, Parsons JT. Motivational interviewing by peer outreach workers: a pilot randomized clinical trial to retain adolescents and young adults in HIV care. AIDS care. 2009;21(7):868–73. 10.1080/09540120802612824 20024744

[36] Volpp KG, Troxel AB, Pauly MV, Glick HA, Puig A, Asch DA, et al A randomized, controlled trial of financial incentives for smoking cessation. New England Journal of Medicine. 2009;360(7):699–709. 10.1056/NEJMsa0806819 19213683

[37] Haukoos JS, Witt MD, Coil CJ, Lewis RJ. The effect of financial incentives on adherence with outpatient human immunodeficiency virus testing referrals from the emergency department. Academic emergency medicine. 2005;12(7):617–21. 15995093 10.1197/j.aem.2005.02.016

[38] Keller SC, Yehia BR, Momplaisir FO, Eberhart MG, Share A, Brady KA. Assessing the Overall Quality of Health Care in Persons Living with HIV in an Urban Environment. AIDS patient care and STDs. 2014;28(4):198–205. 10.1089/apc.2014.0001 24654969 PMC3985506

[39] Gardner LI, Metsch LR, Anderson-Mahoney P, Loughlin AM, Del Rio C, Strathdee S, et al Efficacy of a brief case management intervention to link recently diagnosed HIV-infected persons to care. Aids. 2005;19(4):423–31. 15750396 10.1097/01.aids.0000161772.51900.eb

[40] Kirsten I, Sewangi J, Kunz A, Dugange F, Ziske J, Jordan-Harder B, et al Adherence to combination prophylaxis for prevention of mother-to-child-transmission of HIV in Tanzania. PloS one. 2011;6(6):e21020 10.1371/journal.pone.0021020 21695214 PMC3112206

[41] Mepham S, Zondi Z, Mbuyazi A, Mkhwanazi N, Newell M. Challenges in PMTCT antiretroviral adherence in northern KwaZulu-Natal, South Africa. AIDS care. 2011;23(6):741–7. 10.1080/09540121.2010.516341 21293987

[42] Nassali M, Nakanjako D, Kyabayinze D, Beyeza J, Okoth A, Mutyaba T. Access to HIV/AIDS care for mothers and children in sub-Saharan Africa: adherence to the postnatal PMTCT program. AIDS care. 2009;21(9):1124–31. 10.1080/09540120802707467 20024771

[43] Duff P, Kipp W, Wild TC, Rubaale T, Okech-Ojony J. Barriers to accessing highly active antiretroviral therapy by HIV-positive women attending an antenatal clinic in a regional hospital in western Uganda. Journal of the International AIDS Society. 2010;13(1):37. 20863399 10.1186/1758-2652-13-37 PMC2954932

[44] Turner BJ, Laine C, Cosler L, Hauck WW. Relationship of Gender, Depression, and Health Care Delivery With Antiretroviral Adherence in HIV‐infected Drug Users. Journal of General Internal Medicine. 2003;18(4):248–57. 12709091 10.1046/j.1525-1497.2003.20122.x PMC1494846

[45] Panel on Antiretroviral Guidelines for Adults and Adolescents. Guidelines for the use of antiretroviral agents in HIV-1-infected adults and adolescents. Department of Health and Human Services. December 1, 2009; 1–161. Available at http://www.aidsinfo.nih.gov/ContentFiles/AdultandAdolescentGL.pdf. Accessed July 9, 2014.

