# Supplementary material for: Time of HIV Diagnosis and Engagement in Prenatal Care Impact Virologic Outcomes of Pregnant Women with HIV
Source: PLoS One. 2015 Jul 1;10(7):e0132262. doi: 10.1371/journal.pone.0132262 (PMC4489492; doi:10.1371/journal.pone.0132262)
Supplement: S1 Table — 708 (84.69%) mother-to-child pairs received ART during pregnancy. **VL = viral load. 437 (52.27%) mother-to-child pairs achieved viral suppression at delivery. The HIV VL > 400 copies/ml includes 12.44% missing HIV VL. The VL were measured during pregnancy and up to 30 days postpartum. † Adequacy of prenatal care was measured using the Kessner Index which takes in account timing of entry in prenatal care, the number of prenatal visits and the gestational age at delivery. It is a validated measure of quality of prenatal care. (DOCX) [file pone.0132262.s001.docx]

| **S1 Table.** Demographic, Behavioral and Quality of Care Indicators by Receipt of ART During Pregnancy and Viral Suppression at Delivery among Women Infected  with HIV, Enhanced Perinatal Surveillance Project, Philadelphia, 2005-2013. | **Total, n (%)**  **n=836** | **Receipt of ART*,**  **n (%)** | **No ART,**  **n (%)** | ***P* value** |  | **HIV VL**  **≤400 copies/ml,**  **n (%)**** | **HIV VL**  **> 400 copies/ml,**  **n (%)** | ***P* value** |
| --- | --- | --- | --- | --- | --- | --- | --- | --- |
| **Demographic/Behavioral** |  |  |  |  |  |  |  |  |
| **Age (years)** |  |  |  | 0.14 |  |  |  | 0.93 |
| 16-24 | 214 (25.6) | 190 (26.8) | 24 (18.7) |  |  | 110 (25.2) | 104 (26.1) |  |
| 25-34 | 431 (51.6) | 361 (51.0) | 70 (54.7) |  |  | 228 (52.2) | 203 (50.9) |  |
| ≥35 | 191 (22.8) | 157 (22.2) | 34 (26.6) |  |  | 99 (22.6) | 92 (23.1) |  |
| **Race/Ethnicity** |  |  |  | 0.62 |  |  |  | 0.87 |
| White, non-Hispanic | 107 (12.8) | 64 (9.0) | 8 (6.2) |  |  | 39 (8.9) | 33 (8.3) |  |
| Black, non-Hispanic | 661 (79.1) | 557 (78.7) | 104 (81.3) |  |  | 346 (79.2) | 315 (78.9) |  |
| Hispanic or Latino | 68 (8.1) | 56 (7.9) | 12 (9.4) |  |  | 36 (8.2) | 32 (8.0) |  |
| Other | 35 (4.2) | 31 (4.4) | 4 (3.1) |  |  | 16 (3.7) | 19 (4.8) |  |
| **Insurance** |  |  |  | 0.72 |  |  |  | 0.05 |
| Public | 650 (77.7) | 547 (77.3) | 103 (80.5) |  |  | 325 (74.4) | 325 (81.4) |  |
| Private | 97 (11.6) | 84 (11.9) | 13 (10.2) |  |  | 58 (13.3) | 39 (9.8) |  |
| Uninsured | 89 (10.6) | 77 (10.9) | 12 (9.4) |  |  | 54 (12.4) | 35 (8.8) |  |
| **Drug Use During Pregnancy** |  |  |  | <0.001 |  |  |  | 0.005 |
| Yes | 191 (22.8) | 140 (19.8) | 51 (39.8) |  |  | 83 (19.0) | 108 (27.1) |  |
| No | 645 (77.1) | 568 (80.2) | 77 (60.2) |  |  | 354 (81.0) | 291 (72.9) |  |
|  |  |  |  |  |  |  |  |  |
| **Quality of Care Indicators** |  |  |  |  |  |  |  |  |
| **Adequacy of Prenatal Care†** |  |  |  | <0.001 |  |  |  | <0.001 |
| Adequate | 325 (38.9) | 309 (43.6) | 16 (12.5) |  |  | 196 (44.8) | 129 (32.3) |  |
| Intermediate | 319 (38.2) | 296 (41.8) | 23 (18.0) |  |  | 179 (41.0) | 140 (35.1) |  |
| Inadequate | 192 (23.0) | 103 (14.5) | 89 (69.5) |  |  | 62 (14.2) | 130 (32.6) |  |
| **Timing of HIV Diagnosis** |  |  |  | <0.001 |  |  |  | <0.001 |
| Before Pregnancy | 625 (74.8) | 557 (78.7) | 68 (53.1) |  |  | 349 (79.9) | 276 (69.2) |  |
| During Pregnancy | 211 (25.3) | 151 (21.3) | 60 (46.9) |  |  | 88 (20.1) | 123 (30.8) |  |
| **Birth Year** |  |  |  | <0.001 |  |  |  | <0.001 |
| 2005-2007 | 346 (41.4) | 276 (39.0) | 70 (54.7) |  |  | 123 (28.1) | 223 (55.8) |  |
| 2008-2010 | 308 (36.8) | 264 (37.3) | 44 (34.4) |  |  | 189 (43.2) | 119 (29.8) |  |
| 2011-2013 | 182 (21.8) | 168 (23.7) | 14 (10.9) |  |  | 125 (28.6) | 57 (14.3) |  |
